# Dynamics of *Bemisia tabaci* biotypes and insecticide resistance in Fujian province in China during 2005–2014

**DOI:** 10.1038/srep40803

**Published:** 2017-01-23

**Authors:** Feng-Luan Yao, Yu Zheng, Xiao-Yan Huang, Xue-Ling Ding, Jian-Wei Zhao, Nicolas Desneux, Yu-Xian He, Qi-Yong Weng

**Affiliations:** 1 Institute of Plant Protection, Fujian Academy of Agricultural Sciences, Fuzhou 350013, China; 2 Fujian Key Laboratory for Monitoring and Integrated Management of Crop Pests, Fuzhou 350001, China; 3 Provincial Station of Plant Protection and Quarantine, Fujian Provincial Department of Agriculture, Fuzhou 350001, China; 4 INRA (French National Institute for Agricultural Research), Univ. Nice Sophia Antipolis, CNRS, UMR 1355-7254 Institut Sophia Agrobiotech, 06903, Sophia-Antipolis, France

## Abstract

The whitefly *Bemisia tabaci* (Gennadius) is an important agricultural insect pest worldwide. The B and Q biotypes are the two most predominant and devastating biotypes prevalent across China. However, there are few studies regarding the occurrence of the Q biotype in Fujian Province, China, where high insecticide resistance has been reported in the B biotype. Differences in some biological characteristics between the B and Q biotypes, especially insecticide resistance, are considered to affect the outcome of their competition. Extensive surveys in Fujian revealed that the B biotype was predominant during 2005–2014, whereas the Q biotype was first detected in some locations in 2013 and widely detected throughout the province in 2014. Resistance to neonicotinoids (that have been used for more than 10 years) exhibited fluctuations in open fields, but showed a continual increasing trend in protected areas. Resistance to lambda-cyhalothrin, chlorpyrifos, and abamectin exhibited a declining trend. Resistance to novel insecticides, such as nitenpyram, pymetrozine, sulfoxaflor, and cyantraniliprole, in 2014 was generally below a moderate level. A decline in insecticide resistance in the B biotype and the rapid buildup of protected crops under global temperature increase may have promoted the establishment of the Q biotype in Fujian.

The whitefly *Bemisia tabaci* (Gennadius) (Hemiptera: Aleyrodidae) has been one of the most devastating and economically important agricultural insect pests worldwide in recent decades^1^. It is a phloem-feeder and inflicts damage by direct feeding, secreting honeydew that triggers sooty mold, and by acting as a vector to plant viruses, especially geminivirus^1^.

*Bemisia tabaci* has long been considered a complex species and the term “biotype” has been used to designate whitefly populations^1^,^2^. However, it was recently considered a cryptic species complex, involving at least 24 morphologically indistinguishable species^1^. In this study, however, we use the term “biotype” for consistency with most previous literature. The B and Q biotypes, recently named as Middle East-Asia Minor 1 and Mediterranean, respectively, are the two most predominant, damaging and invasive biotypes worldwide. In the past two decades, the B biotype spread into at least 54 countries, followed by the invasion of the Q biotype, which has been found in at least 10 countries^1^. These two biotypes differ in a range of biological characteristics, including host plant range and adaptability, ability to transmit plant virus, copulation efficiency, composition of harbored symbionts, and expression of resistance to heat shock and insecticide^3^,^4^,^5^,^6^,^7^,^8^,^9^,^10^,^11^,^12^,^13^,^14^. These differences contribute to the competitive outcomes between the two biotypes in various habitats. The B biotype is more adapted to open fields, whereas the Q biotype is more competitive in protected agricultural facilities^15^,^16^.

In China, *B. tabaci* was first recorded in 1949, but did not cause significant damage until the mid-1990s^17^. However, in 2000, *B. tabaci* caused nationwide concern, when the notorious invasive biotype B largely supplanted the indigenous whitefly species and plagued many provinces where it caused serious yield losses in many crops^18^. Biotype Q was first found in 2003 in Kunming, Yunnan Province, where the International Horticultural Exposition was held and this may have been the center of the invasion^19^. Similarly, biotypes B and Q were first detected and found in large numbers in cities where foreign trade, including ornamentals, vegetables, and fruits, was thriving. These two biotypes have now colonized most parts of the country, and the Q biotype, which followed the invasion of the B biotype, is considered the predominant biotype in China^20^,^21^. However, the whitefly population in the Fujian Province is dominated by B biotype^20^, and we could not find any new reference to the Q biotype in Fujian, except for Wang *et al*.^22^, wherein it is stated that the whitefly was found in a suburb of Fuzhou City, the provincial capital of Fujian, and Zhangzhou City, where the vegetable and flower industry is flourishing.

Whitefly control depends heavily on insecticides because of their easy application, quick action, and high efficacy. However, repeated spray applications result in the overuse of insecticides and induce various issues e.g. environmental pollution and impact on non-target organisms^23^,^24^ and the selection of resistant pest populations^25^,^26^,^27^. Whiteflies have been reported to develop resistance to a wide range of insecticides, including conventional ones such as organophosphates, carbamates, pyrethroids, and novel ones, such as neonicotinoids and insect growth regulators^15^,^17^,^22^,^28^. Insecticide resistance in whiteflies lowered the control efficacy of commonly used insecticides and accelerated the need for new insecticide chemistries. Nitenpyram and pymetrozine were both recommended by the National Agro-Tech Extension and Service Center (NATESC) in China as candidate substitutes for conventional, highly hazardous insecticides in 2009^27^,^29^. Cyantraniliprole and sulfoxaflor are the newly registered insecticides against *B. tabaci* that are available.

We have monitored the biotype and insecticide resistance status of *B. tabaci* in Fujian since 2005, and some findings of two experiments conducted in 2005 and 2009 were released by He *et al*.^30^ and Zheng *et al*.^31^. The development of protected agriculture has been advocated and supported by the government of Fujian Province since 2009, and substantial advances have been achieved in recent years, especially since 2013, when the government greatly increased subsidies. Extensive surveys across the entire province were conducted in 2011, 2013, and 2014. Through comprehensive analysis of data over a 10-year period, the main goals of the present study were (1) to reveal the dynamics of biotypes in different habitat types (open field or protected areas); (2) to determine resistance trends against some common insecticides that have been used for more than 10 years in Fujian and the resistance status toward some novel insecticides recently appearing on the market; and (3) to discuss factors impacting the shift in biotype composition.

## Results

### Biotype dynamics during 2005–2014

**Figure 1:**
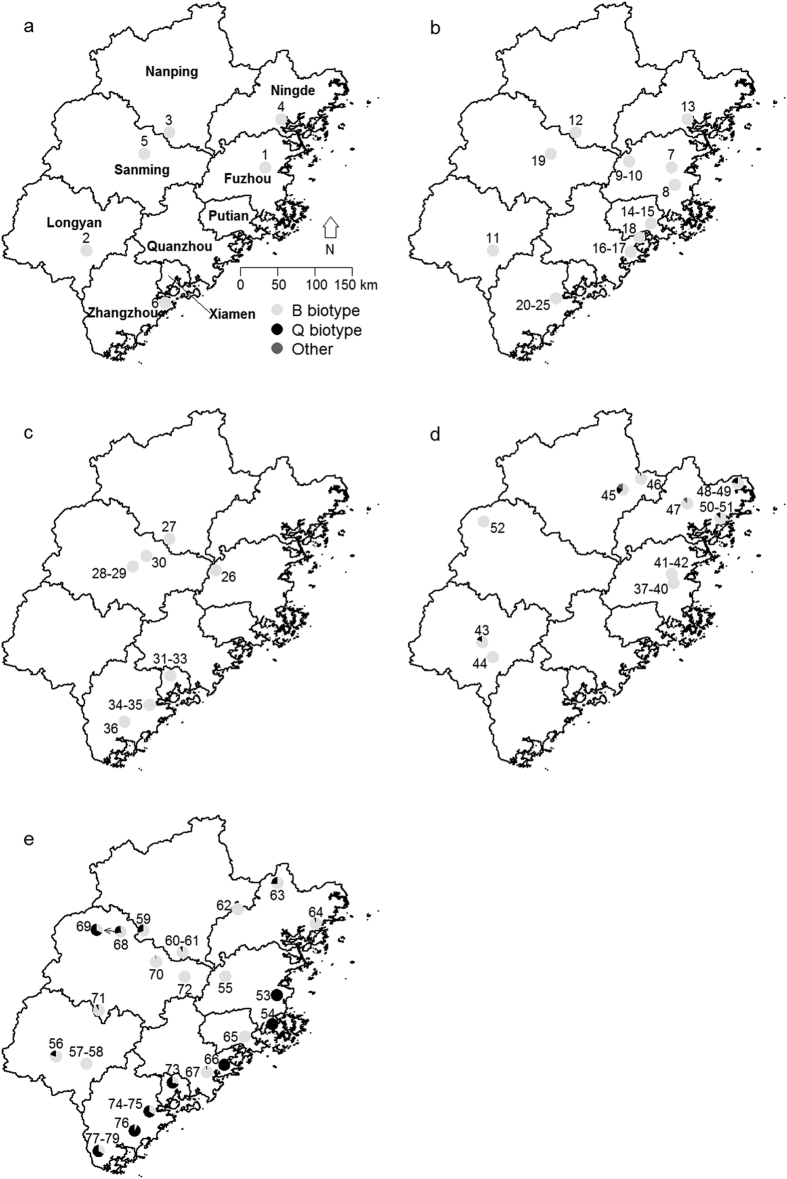
Distribution of *B. tabaci* biotype in Fujian Province from 2005 to 2014. (**a**) 2005; (**b**) 2009; (**c**) 2011; (**d**) 2013; (**e**) 2014. Numbers on the map correspond to population ID numbers in Table 2. Data for 2005 and 2009 partially derived from He *et al*.^30^ and Zheng *et al*.^31^, respectively. Maps were created by using the R statistical program version 2.15.2 65 with four add-on packages ‘maps’^66^, ‘maptools’^67^, ‘ggplot2’^68^ and GISTools^69^. The data for creating the base map of Fujian Province, China was obtained from the Global Administrative Areas (GADM) database (Version 2.8, http://www.gadm.org/).

**Figure 2:**
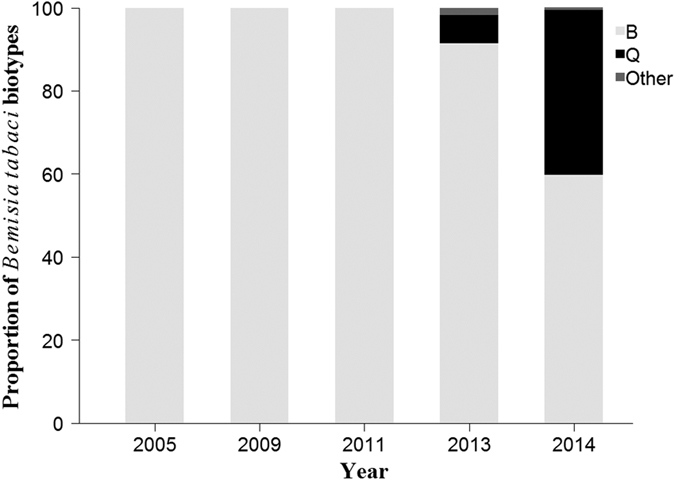
Proportion of *Bemisia tabaci* biotypes in Fujian Province during 2005–2014. Data for 2005 and 2009 derived from He *et al*.^30^ and Zheng *et al*.^31^, respectively.

**Figure 3:**
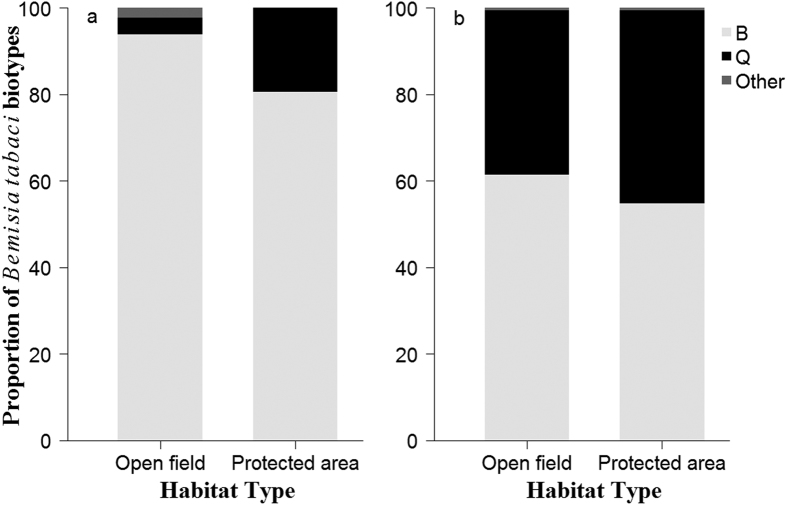
Proportion of *Bemisia tabaci* biotypes in different habitat types in Fujian Province in 2013 (**a**) and 2014 (**b**).

Distribution and dynamics of *B. tabaci* collected from various areas of Fujian from 2005 to 2014 are shown in Figs 1, 2 and 3. A total of 1,958 whitefly individuals from 79 populations in terms of year, site, and host plant were identified as the B or Q biotype, and others. The B biotype was the only biotype detected during the first three sampling years and the predominant biotype in 2013 and 2014, regardless of habitat type. However, the Q biotype was first found in parts of Ningde, Nanping, and Longyan in 2013, and widely distributed throughout the province in 2014. Its percentage in terms of total whiteflies collected increased from 6.8% in 2013 to 39.7% in 2014. Although none of the 16 populations sampled in 2013 were exclusively of the Q biotype, five of the 27 sampled populations in 2014 were. For those five populations, three occurred in open fields (ID numbers: 53, 77, and 78) and two in protected areas (ID numbers: 54 and 66). The number and proportion of whitefly populations that included only the B biotype decreased from 10 and 62.5% in 2013 to nine and 33.3% in 2014, respectively. There were four populations where the B and Q biotypes co-occurred in 2013 (ID numbers: 43, 45, 46, and 48), whereas in 2014 the number rose to 13 (ID numbers: 56, 59, 60, 63, 64, 67–69, 71, and 73–76), and most of these populations of mixed B and Q biotypes were found in open fields.

### Resistance to insecticides in use since 2005

**Figure 4:**
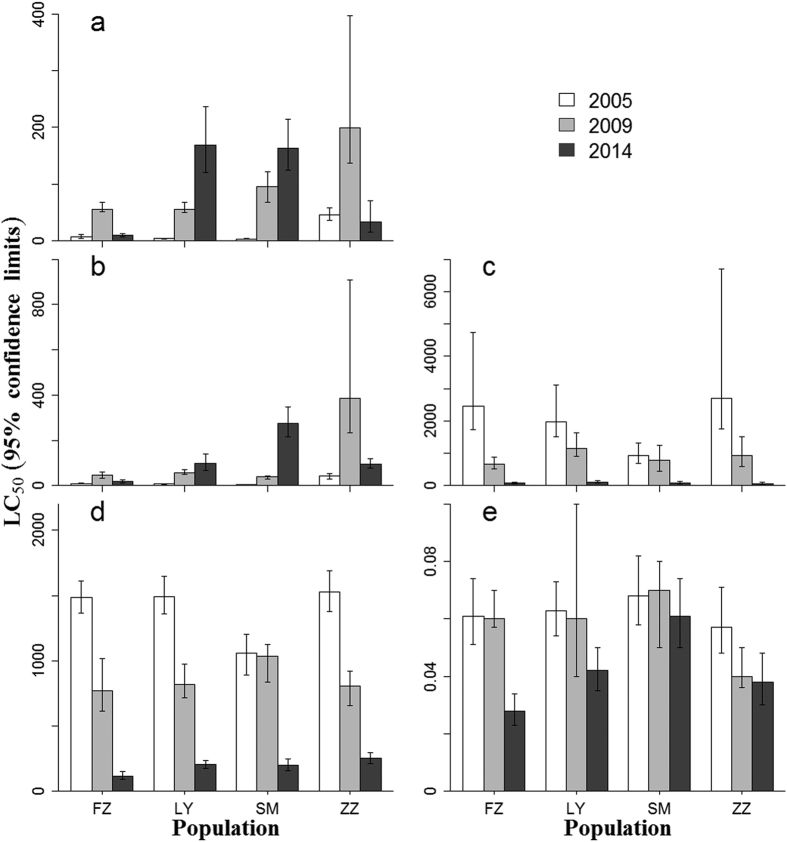
LC_50_ values for different insecticides (**a**) imidacloprid; (**b**) thiamethoxam; (**c**) lambda-cyhalothrin; (**d**) chlorpyrifos; and (**e**) abamectin) against populations of *Bemisia tabaci* collected from different locations in Fujian, China, in 2005, 2009, and 2014. Whitefly populations of LY and SM collected in 2014 were sampled from protected areas; others were sampled from open fields. Whitefly populations SM and ZZ collected in 2014 were coexisting populations of B and Q biotypes, but dominated by Q; others were the B biotype. LC_50_ values for each insecticide in 2005 and 2009 derived from He *et al*.^30^ and Zheng *et al*.^31^, respectively.

The toxicity data of six commonly used insecticides against *B. tabaci* were monitored in 2005, 2009, and 2014 (Fig. 4). LC_50_ values of imidacloprid and thiamethoxam were significantly higher in 2009 than in 2005, and the decline in the LC_50_ values in 2014 relative to 2009 was observed in open field populations, such as FZ and ZZ, but not in protected area populations of LY and SM. For these, a continuing increase in the LC_50_ values was found. However, lambda-cyhalothrin and chlorpyrifos LC_50_ values attained their highest value in 2005, followed by lower values in 2009, and their lowest in 2014, regardless of where the populations originated. For abamectin, though significant declines in the LC_50_ values were found in some populations in 2014 relative to that of 2005, the value generally did not change substantially from year to year.

### Resistance to insecticides in use since 2009

Toxicity of novel insecticides against *B. tabaci* is presented in Table 1. All of the four collected whitefly populations exhibited very low resistance to nitenpyram and from very low to low resistance to pymetrozine. The resistance factors for the tested populations for nitenpyram were no more than 6-fold, and for pymetrozine were no more than 12-fold. Although low and moderate resistance was found to cyantraniliprole, none to moderate resistance was detected to sulfoxaflor. The resistance factors to nitenpyram and sulfoxaflor were not always higher in open field populations than in protected area populations. However, for pymetrozine and cyantraniliprole, protected area populations always exhibited higher resistance factors than open field populations.

## Discussion

Since the first detection of the Q biotype in the Yunnan Province in 2003^19^, the Q biotype has subsequently been found and become the prevalent biotype in the Yangtze River Valley^19^,^32^,^33^,^34^,^35^ and on crops in most locations in China^21^, although the south and southeastern coastal areas of China are dominated by the B biotype^20^. Our research also revealed that the B biotype was always the predominant biotype in Fujian during 2005–2014. In addition, the Q biotype was first detected in some areas in 2013 and widely detected throughout the province in 2014. Wang *et al*.^22^ reported finding the Q biotype in Fuzhou and Zhangzhou cities on sampled cauliflower in October 2008. Although another record relevant to the detection of the Q biotype in Fujian occurs in GenBank (accession number FJ594434), we could not find any descriptions in the paper published by Teng *et al*.^36^. Regardless, it appears that the Q biotype invaded Fujian, particularly in areas where the development of agriculture was advanced. However, population establishment of the Q biotype may have not been successful initially. We re-sampled whiteflies in the fields where Wang *et al*.^22^ had in 2009, but we failed to detect the Q biotype using the specific mtCOI primers used in the present study (see section 2.2) or mtCOI sequencing as in our previous research^31^, implying that the Q biotype previously invaded Fujian, but failed to colonize it before 2012. Multiple secondary introductions, however, may be an important prerequisite for the establishment of the Q population in the fields^37^.

A stronger resistance to insecticide is often regarded as a major cause for the establishment and the dominance of the Q biotype^38^,^39^, although the B biotype has been shown to be more competitive than the Q biotype under untreated conditions^5^,^40^,^41^. However, if the B biotype could rapidly evolve a high resistance, it would supplant the Q biotype even when repeatedly exposed to insecticides^41^. Moreover, the B biotype, being slightly greater in number (30 *vs.* 10), was found to displace the Q biotype in six generations when exposed to a low dose of neonicotinoid insecticide^39^. Thus, the B biotype, with high resistance and abundance, would impede the establishment of the Q biotype. This is in agreement with the fact that only the B biotype was sampled before 2012 by our research team and Pan *et al*.^21^ and the finding that the Q biotype was rare in Fujian, where high levels of resistance to various insecticides in the B biotype have been found^30^,^31^. Similarly, Crowder *et al*.^41^ concluded it was the ability of the B biotype to rapidly evolve resistance to insecticides in the United States but not Israel that resulted in the exclusion of the Q biotype from the field in the United States^42^, but co-occurrence with and dominance over the B biotype in Israel^5^,^15^,^43^. Moreover, the shift in the predominant biotype from the Q to B in 2003–2012 may have partially resulted from an increase in resistance to insecticides of the B biotype in Israel^38^.

The decrease in abundance, as well as the resistance of the B biotype to some extent, facilitated the establishment of the Q biotype. Broadly, the abundance of *B. tabaci* in open fields sharply increased in early 2000s and maintained a high level until approximately 2010, and then gradually declined to a low level in Fujian. The population decline lowered the demand for insecticide application in recent years, which resulted in a drop in resistance in overall (Fig. 4) from 2005 to 2014 and low-level resistance, especially in open field populations in 2014 (Table 1). The Q biotype, being more resistant than the B biotype, superseded the B biotype when they were at the same initial number and exposed to a low concentration of a neonicotinoid insecticide^39^.

Another possible explanation for the establishment of the Q biotype in Fujian is the rapid buildup of protected agriculture across the province under climate warming. The Q biotype is more tolerant to heat stress than the B biotype^4^,^6^, which gives the Q biotype an advantage over the B biotype in environments with high temperature. The Q biotype that invaded Fujian must have encountered the B biotype under extremely hot temperatures in recent years. On one hand, rising temperatures characterized the recent and current climate^44^. In the summer of 2013, an extremely unusual heat wave hit central and eastern China. The average daily mean temperature, daily maximum temperature, daily minimum temperature, and number of days with daily maximum temperature that reached or exceeded 35 °C were all greater in 2013 than in 1960–2012^45^. Moreover, a continuing warming was observed in Fujian in the summer of the next year, when the average seasonal temperature increased from 27.4 °C in 2013 to 28.0 °C in 2014^46^. On another hand, the development of protected agriculture was encouraged and subsidized by the government since 2009, and its advancement was accelerated since 2013 when financial assistance increased substantially. Thus, high temperatures ≥35 °C, which appear in the open fields and often exist in protected areas, may have contributed to the results in this paper, specifically the colonization of the Q biotype and its proportional increase in whitefly population. Chu *et al*.^37^ demonstrated that wide use of the protected agriculture in northern China promoted the colonization of the Q biotype in agricultural fields.

Although it is now dominated by the B biotype, a continuous increase of the Q biotype in protected areas in Fujian is probable. Kontsedalov *et al*.^15^ and Hsieh *et al*.^16^ both found that the B biotype dominated crops in open fields and the Q biotype dominated crops in protected agricultural areas. However, Kontsedalov *et al*.^15^ speculated that the high fitness rate for the B biotype and the inefficiency of insecticide control contributed to the predominance of the B biotype in open fields, whereas Hsieh *et al*.^16^ regarded temperature as the main impact factor. Although resistance to repeated insecticide spraying and high temperature stress contribute to the domination of the Q biotype in protected areas^15^, different kinds of insecticides in use may influence the *B. tabaci* biotype composition^16^. Other than insecticide, habitat type, and climate, the shift in biotype composition may also result from the infestation of *B. tabaci* by its symbionts and viruses^11^,^12^,^38^.

Based on the resistance data tested in 2005, 2009, and 2014, we can infer that, in general, field populations of *B. tabaci* resistant to neonicotinoids underwent an increase in the first several years and a reduction during the subsequent 10-year period. Yearly population dynamics of *B. tabaci* in Fujian, as mentioned above, may reflect the variance of the resistance. Although a shift in the biotype composition for the ZZ population was detected in 2014, resistance may not be the reason for the decline because the Q biotype was more resistant to neonicotinoids than the B biotype^9^,^39^,^41^. However, neonicotinoid resistance in protected areas, such as greenhouses, net houses, plastic houses, etc., was completely different. Although a drop in neonicotinoid resistance was observed for open field populations in 2014, a continuous rise was found for LY and SM populations, both from plastic houses. The former contained the B biotype only, whereas the latter consisted of mixed B and Q biotypes, but was dominated by the Q biotype. Thus, *B. tabaci* in protected areas will increase in resistance regardless of biotype composition, because *B. tabaci* is prone to outbreak in these areas, and both B and Q biotypes have the capacity to evolve strong resistance to insecticides, including neonicotinoids^22^,^31^,^47^.

In contrast to neonicotinoids, resistance of *B. tabaci* from open field and protected area to lambda-cyhalothrin and chlorpyrifos constantly decreased in the three test years, implying a declining trend from 2005 to 2014. The replacement with new chemicals, such as neonicotinoids, with novel modes of action relative to conventional insecticides, such as pyrethroids, organophosphates, and carbamates, will likely contribute to a dramatic decline in resistance to conventional insecticides^28^,^48^. Insecticide resistance in *B. tabaci* was demonstrated to be positively correlated with the frequency of insecticide application^49^. A shift from synergized pyrethroids to buprofezin and pyriproxyfen in *B. tabaci* control resulted in a significant reduction in pyrethroid use and recovered the susceptibility to synergized pyrethroids^50^. Similarly, rotation of old and new chemicals that prevents or delays the onset of resistance in whiteflies may have resulted in the susceptibility to lambda-cyhalothrin during 1992–2000 and chlorpyrifos during 1992–2007, whereas these two insecticides were commonly used in Pakistan^28^,^48^. In the present study, we also found that no resistance existed to abamectin. Abamectin appears efficacious in controlling not only the B biotype, but also the Q biotype, as clarified by Wang *et al*.^22^ and Xie *et al*.^51^.

Nitenpyram and pymetrozine have been recommended for use by the agricultural department since 2009, when high levels of resistance to commonly used insecticides in Fujian Province were detected. Although high or very high levels of resistance to imidacloprid and thiamethoxam were detected in protected areas (Fig. 4), very low and low levels of resistance to nitenpyram and pymetrozine were found there, indicating these two insecticides could still be recommended for use.

Cyantraniliprole and sulfoxaflor were temporally registered in China in 2012 and 2013, respectively, and their commercial products were available on the market in 2013. Although cyantraniliprole, being an anthranilic diamide insecticide, acts exclusively on the ryanodine receptor in insects, it was thought to be highly efficacious against *B. tabaci*^52^,^53^. However, high LC_50_ values observed in open field (35.95–64.22 mg A.I. L^−1^) and protected areas (103.77–118.41 mg A.I. L^−1^) relative to the recommended field concentration of 66.66 mg A.I. L^−1^ indicated a potentially high risk in using cyantraniliprole to control *B. tabaci*. Xie *et al*.^51^ also reported the inefficiency of cyantraniliprole against adult *B. tabaci*, although it was highly effective against eggs and larvae.

Sulfoxaflor targets insect nicotinic acetylcholine receptors (nAChRs) and functions discriminately with other insecticides acting at nAChRs, like neonicotinoids^54^. A lack of cross-resistance between sulfoxaflor and neonicotinoids has been demonstrated in *B. tabaci* and *Trialeurodes vaporariorum*^55^,^56^. In comparison with the recommended field concentration of 73.3 mg A.I. L^−1^, a considerably low LC_50_ value (0.64–12.61 mg A.I. L^−1^) along with slope values less than 1.5 were found in the four populations, suggesting that sulfoxaflor may be an important new tool for whitefly control.

## Conclusions

The Q biotype was first detected in some areas in 2013, followed by frequent detection throughout Fujian Province in 2014. However, the whitefly population was predominated by the B biotype during 2005–2014, although the Q biotype appeared to be increased in proportion not only in open fields but also in protected areas. The abundant B biotype, with high levels of resistance, may prevent the colonization of the Q biotype even in cropping systems where insecticides were overused, although the Q biotype was thought to be more resistant. The establishment of the Q biotype, however, might have been facilitated by the decline in the abundance of the B biotype which resulted in a drop in insecticide resistance, as well as a warming environment, that is, the rapid buildup of protected agriculture in the context of global temperature rise.

Resistance to neonicotinoids used for more than 10 years exhibited fluctuations in open fields, but a continual increase in protected areas. Conversely, a continuous decline was observed for conventional insecticides, such as pyrethroids and organophosphates. Resistance variance reflects the change in intensity of insecticide utilization. Application reduction, therefore, contributes to some extent to the decline in resistance, which indicates the importance of rational rotation in insecticide resistance management. No resistance was found to abamectin during 2005–2014 demonstrating that it is a powerful insecticide against *B. tabaci*, regardless of biotype. Four novel insecticides, nitenpyram, pymetrozine, and sulfoxaflor, are suitable for controlling both biotypes of *B. tabaci* in open fields and protected agricultures, although cyantraniliprole should be used prudently. Judicious rotation of conventional and novel insecticides should be adopted in whitefly resistance management.

## Material and Methods

### Whitefly collection

Samples in open field and protect area populations of whiteflies were collected from various crops, but primarily from vegetables in intensive agricultural regions throughout Fujian Province, China in 2005, 2009, 2011, 2013, and 2014 (Table 2 and Fig. 1). Adult whiteflies were sampled using a simple mouth suction apparatus. Samples used for resistance bioassays were first anesthetized by CO_2_ and then transferred into plastic ventilated petri dishes (Φ = 3.5 cm) where cotton leaves, placed with their adaxial surface downwards onto the agar, were provided for whitefly population maintenance. For those used for biotype determination, samples were either anesthetized by CO_2_, stored in 95% ethanol, and taken back to the laboratory where they were frozen at −20 °C or host plant leaves, along with whiteflies, were placed in plastic bags, and then taken back to the laboratory and frozen at −20 °C.

### Biotype determination

For whitefly samples collected in 2005 and 2009, direct sequencing of the mitochondrial cytochrome oxidase I (mtCOI) gene was used to identify the biotype of whitefly individuals. The methodology followed He *et al*.^57^. Briefly, whitefly DNA was extracted from individual adults following the method of Luo *et al*.^58^. The primers C1-J-2195 (5′-TTGATTTTTTGGTCATCCAGAAGT-3′) and L2-N-3014 (5′-TCCAATGCACTAATCTGCCATATTA-3′) described in Frohlich *et al*.^59^ were used for PCR amplification. Total reaction volumes of 30 μL were used with a final concentration involving 1.5 U of Taq polymerase, 2 mg mL^−1^ BSA, 0.25 mM of dNTPs, 2.5 mM MgCl_2_, 75 ng C1-J-2195, 75 ng L2-N-3014, and 4 μL of DNA. Amplification was conducted in an Eppendorf thermocycler using the following parameters: 94 °C for 5 min, followed by 35 cycles at 94 °C for 1 min, 50 °C for 1 min, 72 °C for 1 min, and a final extension at 72 °C for 5 min. PCR products were electrophoresed in 1.2% agarose gel and purified by Promega DNA gel extraction kit and sequenced directly in two strands by Invitrogen (Shanghai, China). Biotype was determined for 24 individual adults for each population.

The biotype of whiteflies collected in 2011, 2013, and 2014 was determined individually through the amplification of the mtCOI gene by PCR with the specific primers described in Shatters *et al*.^60^, that is, for the B biotype forward primer, 5′-CTAGGGTTTATTGTTTGAGGTCATCATATATTC-3′, and reverse primer, 5′-AATATCGACGAGGCATTCCCCCT-3′; for the Q biotype, the forward primer, 5′-CTTGGTAACTCTTCTGTAGATGTGTGTT-3′, and reverse primer, 5′-CCTTCCCGCAGAAGAAATTTTGTTC-3′. PCR reaction was performed in a volume of 20 μL containing 4 μL double distilled water, 10 μL 2 × Long master mix (TianGen Biotech, Beijing, China), 0.5 μL (10 μM) forward primer for both biotypes, 0.5 μL (10 μM) reverse primer for both biotypes, and 4 μL template DNA. PCR procedure consisted of an initial denaturation of 94 °C for 2 min, followed by 35 cycles at 94 °C for 0.5 min, 64 °C for 1 min, 72 °C for 1 min, and a final extension at 72 °C for 7 min. PCR products were separated by electrophoresis in a 1.2% agarose gel. *Bemisia tabaci* biotypes were easily identified based on the criterion of 478 bp for the B biotype and 303 bp for the Q biotype^21^. A total of 13–57, but typically 24 individual adults were identified for each population.

### Insecticides

The commercial formulated insecticides used for leaf-dip bioassays were: imidacloprid (imidacloprid 10% WP, Shenyang Sciencreat Chemicals Co., Ltd, China); thiamethoxam (Actara 25% WG, Syngenta Crop Protection, Switzerland); nitenpyram (Cishen 10% SL, Guangxi Kangsaide Biotechnology Co., Ltd., China); chlorpyrifos (Lorsban 40% EC, Dow AgroSciences, USA); lambda-cyhalothrin (Jingbiao 2.5% EC, Syngenta Crop Protection, Switzerland); pymetrozine (Feineng 50% WP, Shaanxi Sunger Road Bio-Science Co., Ltd., China); abamectin (Yingli 2% CS, Hebei Veyong Bio-Chemical Co., Ltd., China); spirotetramat (Movento 22.4% SC, Bayer CropScience, German); cyantraniliprole (Benevia 10% OD, DuPont, USA); and sulfoxaflor (Transform 22% SC, Dow AgroSciences, USA).

### Bioassays

Samples collected from fields were reared on cotton plants in the laboratory at 25–28 °C, 65 ± 5% RH, and 14:10 h (L:D) photoperiod, and the F_1_ generation adults were used for bioassays. Leaf dip bioassays with *B. tabaci* adults followed the methodology of He *et al*.^61^. Cotton leaf discs (Φ = 3.5 cm) were dipped for 5 s in 5–8 serial dilutions of insecticides or in water as the control. After air drying, leaf discs were placed with their adaxial surface downward onto agar in a petri dish (Φ = 3.5 cm). Twenty-five to thirty-five *B. tabaci* female adults were briefly anesthetized with CO_2_ and gently transferred onto the leaf discs. Each petri dish was then covered with a perforated lid and placed inverted in a growth chamber at 25 ± 1 °C, 65 ± 5% RH, and 14:10 h (L:D) photoperiod. Each insecticide treatment was repeated three times and mortality was recorded after 48 h, with the exception of pymetrozine, which was examined after 96 h.

### Statistical analysis

Data were corrected for control mortality using Abbott’s formula^62^ and analyzed by probit analysis^63^. Significant differences between LC_50_ values were determined by the absence of the overlap of the 95% confidence limits. Resistance factors (RFs) were obtained by dividing LC_50_ values by the corresponding values of the susceptible population (Lab-S) reared in laboratory without exposure to any insecticides since 2008. Based on Ahmad and Arif^64^, resistance level was ranked as none (RF < 2), very low (2 ≤ RF < 11), low (11 ≤ RF < 21), moderate (21 ≤ RF < 51), high (51 ≤ RF < 100), or very high (RF ≥ 100).

Maps were created by using the R statistical program version 2.15.2^65^ with four add-on packages ‘maps’^66^, ‘maptools’^67^, ‘ggplot2’^68^ and GISTools^69^.

## Additional Information

**How to cite this article**: Yao, F.-L. *et al*. Dynamics of *Bemisia tabaci* biotypes and insecticide resistance in Fujian province in China during 2005–2014. *Sci. Rep.* **7**, 40803; doi: 10.1038/srep40803 (2017).

**Publisher's note:** Springer Nature remains neutral with regard to jurisdictional claims in published maps and institutional affiliations.

## Figures and Tables

**Table 1:** Responses of field populations of *Bemisia tabaci* collected in 2014 to new insecticides that were not widely used or available before 2009 in Fujian, China.

Insecticide	ID^*^	Population^†^	*N*	LC_50_ (95% FL) (mg A.I. L^−1^)	Slope ± SE	*χ*^2^	*df*	*P*	Resistance factor	Resistance level
Nitenpyram	—	Lab-S	561	0.66 (0.51–0.85)	1.03 ± 0.11	1.59	4	0.81	1	—
	55	FZ	500	2.61 (1.78–3.82)	1.24 ± 0.15	5.27	4	0.26	3.95	Very low
	57	LY	630	2.34 (1.50–3.63)	1.01 ± 0.11	5.87	5	0.32	3.55	Very low
	69	SM	473	3.91 (2.80–5.48)	1.10 ± 0.15	3.04	3	0.39	5.92	Very low
	75	ZZ	462	1.96 (1.53–2.52)	1.16 ± 0.15	5.57	3	0.13	2.97	Very low
Pymetrozine	—	Lab-S	592	6.14 (4.91–7.69)	1.14 ± 0.11	3.53	4	0.47	1	—
	55	FZ	563	21.43 (16.39–28.02)	0.98 ± 0.11	1.94	4	0.75	3.49	Very low
	57	LY	544	69.25 (56.15–85.41)	1.47 ± 0.13	4.54	4	0.34	11.28	Low
	69	SM	557	25.59 (21.01–31.18)	1.35 ± 0.12	5.81	4	0.21	4.17	Very low
	75	ZZ	518	14.29 (9.27–22.02)	0.73 ± 0.11	0.70	4	0.95	2.33	Very low
Cyantraniliprole	—	Lab-S	537	3.14 (2.27–4.33)	1.02 ± 0.12	2.47	4	0.65	1	—
	55	FZ	625	52.29 (32.64–83.78)	0.88 ± 0.10	8.82	4	0.07	16.65	Low
	57	LY	469	118.41 (90.63–154.69)	1.21 ± 0.15	3.00	3	0.39	37.71	Moderate
	69	SM	494	103.77 (85.48–125.98)	1.48 ± 0.16	2.37	3	0.50	33.05	Moderate
	75	ZZ	584	64.22 (43.36–95.12)	0.78 ± 0.11	2.86	4	0.58	20.45	Low
Sulfoxaflor	—	Lab-S	549	0.40 (0.34–0.46)	1.75 ± 0.13	3.84	4	0.43	1	—
	55	FZ	394	0.64 (0.50–0.83)	1.31 ± 0.17	4.92	3	0.18	1.60	None
	57	LY	398	0.99 (0.72–1.37)	0.90 ± 0.16	1.10	3	0.78	2.48	Very low
	69	SM	738	9.13 (6.80–12.27)	0.88 ± 0.08	2.60	6	0.86	22.83	Moderate
	75	ZZ	451	12.61 (10.17–15.64)	1.34 ± 0.16	1.93	3	0.59	31.53	Moderate

*ID number coincides with that in Table 2. ^†^Lab-S, FZ, and LY were B biotype; SM and ZZ were co-existing populations of the B and Q biotypes, dominated by Q.

**Table 2:** *Bemisia tabaci* populations sampled during 2005–2014 throughout Fujian Province for assessing biotype dynamics and resistance status.

ID	Location			Host plant	Habitat type	Collection date	n^†^
	City	District	Town/Street				
1^*^	Fuzhou	Jin’an	Xindian	Cucumber	Open field	2005/8/4	24
2^*^	Longyan	Xinluo	Hongfan	Cucumber	Open field	2005/8/12	24
3^*^	Nanping	YanPing	Xiqin	Cucumber	Open field	2005/8/21	24
4	Ningde	Jiaocheng	Qidu	Eggplant	Open field	2005/8/7	24
5^*^	Sanming	Meilie	Yangxi	Cucumber	Open field	2005/8/20	24
6^*^	Zhangzhou	Longhai	Gangwei	Eggplant	Open field	2005/8/13	24
7^*^	Fuzhou	Jin’an	Xindian	Cucumber	Open field	2009/9/7	24
8		Minhou	Qingkou	Cucumber	Open field	2009/9/7	24
9		Minqing	Baizhang	Cucumber	Open field	2009/9/2	24
10			Yunlong	Brocoli	Open field	2009/9/2	24
11^*^	Longyan	Xinluo	Hongfan	Cucumber	Open field	2009/9/4	24
12	Nanping	Yanping	Xiqin	Cucumber	Open field	2009/9/1	24
13	Ningde	Jiaocheng	Qidu	Cucumber	Open field	2009/9/6	24
14	Putian	Licheng	Huangshi	Winter melon	Open field	2009/9/4	24
15				Pumpkin	Open field	2009/9/4	24
16	Quanzhou	Hui’an	Luoyang	Sweet potato	Open field	2009/9/4	24
17				Soybean	Open field	2009/9/4	24
18		Quangang	Nanpu	Cabbage	Open field	2009/10/28	24
19^*^	Sanming	Meilie	Yangxi	Eggplant	Open field	2009/9/1	24
20	Zhangzhou	Longhai	Baishui	Sweet potato	Open field	2009/9/3	24
21			Bangshan	Cucumber	Open field	2009/9/3	24
22			Dongyuan	Pumpkin	Open field	2009/9/3	24
23			Fugong	Cucumber	Open field	2009/9/3	24
24^*^			Gangwei	Cucumber	Open field	2009/9/3	24
25			Haicheng	Soybean	Open field	2009/9/3	24
26	Fuzhou	Minqing	Jinsha	Eggplant	Open field	2011/8/14	24
27	Nanping	Yanping	Xiqin	Soybean	Open field	2011/8/15	24
28	Sanming	Sanyuan	Chengdong	Cucumber	Open field	2011/8/16	24
29				Eggplant	Open field	2011/8/16	24
30		Shaxian	Qiujiang	Cucumber	Open field	2011/8/16	24
31	Xiamen	Tong’an	Lianhua	Soybean	Open field	2011/8/20	24
32				Sweet potato	Open field	2011/8/20	24
33				Cucumber	Open field	2011/8/20	24
34	Zhangzhou	Longhai	Bangshan	Soybean	Open field	2011/8/18	24
35			Haicheng	Soybean	Open field	2011/8/18	24
36		Zhangpu	Shiliu	Eggplant	Open field	2011/8/19	24
37	Fuzhou	Cangshan	Chengmen	Sweet potato	Open field	2013/9/26	24
38				Cabbage	Open field	2013/9/26	24
39				Brocoli	Open field	2013/9/26	24
40				Cucumber	Open field	2013/9/26	24
41		Jin’an	Xindian	Eggplant	Open field	2013/9/26	24
42				Cucumber	Open field	2013/9/26	24
43	Longyan	Shanghang	Gutian	Cucumber	Protected area	2013/11/10	24
44		Xinluo	Hongfan	Cucumber	Open field	2013/9/12	24
45	Nanping	Jian’ou	Chuanshi	Sweet potato	Open field	2013/10/9	24
46		Zhenghe	Tieshan	Eggplant	Open field	2013/8/27	24
47	Ningde	Fu’an	Muyang	Soybean	Open field	2013/8/29	24
48		Fuding	Guanling	Eggplant	Protected area	2013/8/29	24
49			Tongshan	Soybean	Open field	2013/8/29	24
50		Xiapu	Yantian	Soybean	Open field	2013/8/28	24
51			Zhouyang	Sweet potato	Open field	2013/8/18	24
52	Sanming	Jianning	Zixi	Sweet potato	Open field	2013/9/6	24
53	Fuzhou	Changle	Shouzhan	Cucumber	Open field	2014/11/12	24
54		Fuqing	Jiangjing	Cucumber	Protected area	2014/11/6	24
55^*^		Minqing	Baizhang	Tomato	Open field	2014/6/6	24
56	Longyan	Shanghang	Gutian	Cucumber	Protected area	2014/11/6	24
57^*^		Xinluo	Hongfan	Tomato	Protected area	2014/9/2	24
58				Eggplant	Protected area	2014/9/2	24
59	Nanping	Shunchang	Yuankeng	Soybean	Open field	2014/8/27	13
60		Yanping	Xiadao	Soybean	Open field	2014/8/27	26
61			Xiqin	Soybean	Open field	2014/8/27	24
62	Ningde	Pingnan	Lingxia	Broccoli	Open field	2014/12/9	24
63		Shouning	Da’an	African daisy	Open field	2014/7/31	26
64		Xiapu	Songcheng	Cabbage	Open field	2014/10/31	24
65	Putian	Licheng	Huangshi	Eggplant	Open field	2014/12/15	24
66	Quanzhou	Hui’an	Luoyang	Hami melon	Protected area	2014/11/20	28
67		Licheng	Fuqiao	Brocoli	Open field	2014/10/30	24
68	Sanming	Jiangle	Guangming	Soybean	Open field	2014/8/28	24
69^*^				Tomato	Protected area	2014/8/28	40
70		Shaxian	Qiujiang	Tomato	Protected area	2014/11/7	24
71		Yong’an	Xiaotao	Tomato	Open field	2014/11/5	24
72		Youxi	Chengguan	Sweet potato	Open field	2014/8/28	24
73	Xiamen	Tong’an	Datong	Eggplant	Open field	2014/12/9	36
74	Zhangzhou	Longhai	Dongyuan	Cabbage	Open field	2014/9/3	36
75^*^		Longhai	Gangwei	Cucumber	Open field	2014/9/3	57
76		Zhangpu	Sui’an	Cherry tomato	Open field	2014/12/12	16
77		Zhao’an	Qiaodong	Eggplant	Open field	2014/10/31	24
78			Xitan	Eggplant	Open field	2014/10/31	24
79				Cucumber	Open field	2014/11/10	24

*Population used for resistance testing. ^†^The number of whiteflies used for biotype determination.
